# Neutralization of B-Cell Activating Factor (BAFF) by Belimumab Reinforces Small Molecule Inhibitor Treatment in Chronic Lymphocytic Leukemia

**DOI:** 10.3390/cancers12102725

**Published:** 2020-09-23

**Authors:** Claudia Tandler, Moritz Schmidt, Jonas S. Heitmann, Julia Hierold, Jonas Schmidt, Pascal Schneider, Daniela Dörfel, Juliane Walz, Helmut R. Salih

**Affiliations:** 1 Clinical Collaboration Unit Translational Immunology, German Cancer Consortium (DKTK) and German Cancer Research Center (DKFZ), Department of Internal Medicine, University Hospital Tuebingen, 72076 Tuebingen, Germany; Claudia.Tandler@med.uni-tuebingen.de (C.T.); Moritz.Schmidt@med.uni-tuebingen.de (M.S.); Jonas.Heitmann@med.uni-tuebingen.de (J.S.H.); Wild.Julia85@googlemail.com (J.H.); Jonas.Schmidt@med.uni-tuebingen.de (J.S.); Daniela.Doerfel@krh.de (D.D.); Juliane.Walz@med.uni-tuebingen.de (J.W.); 2 Department of Biochemistry, University of Lausanne, 1066 Epalinges, Switzerland; Pascal.Schneider@unil.ch; 3 DFG Cluster of Excellence 2180 ‘Image-Guided and Functional Instructed Tumor Therapy’ (iFIT), Eberhard Karls University, 72076 Tuebingen, Germany

**Keywords:** chronic lymphocytic leukemia, B-cell activating factor, BAFF, belimumab, idelalisib, ibrutinib, venetoclax

## Abstract

**Simple Summary:**

Chronic lymphocytic leukemia (CLL) is the most common form of leukemia in Western countries. Despite the substantial progress achieved by the recent introduction of the novel small molecule inhibitors idelalisib, ibrutinib and venetoclax in CLL treatment, therapy resistance occurs frequently and the disease so far remains incurable. In the present study we report that BAFF, a member of the TNF protein family, protects CLL cells from treatment-induced cell death. In turn, the therapeutic effects of idelalisib, ibrutinib and venetoclax can be reinforced by neutralizing BAFF with belimumab, an antibody which presently is clinically approved for treatment of systemic lupus erythematosus. Based on the data presented in this study, a clinical study to evaluate whether drug repurposing of belimumab for BAFF neutralization can serve to improve response to small molecule inhibitor treatment in CLL is in preparation.

**Abstract:**

The introduction of idelalisib, ibrutinib and venetoclax for treatment of chronic lymphocytic leukemia (CLL) has greatly improved long term survival of patients. However, many patients do not achieve complete remission and suffer from development of resistance upon treatment with these small molecule inhibitors. Here we report that the TNF family member B-cell activating factor (BAFF) mediates resistance of CLL cells to idelalisib, ibrutinib and venetoclax by sustaining survival and preventing apoptosis of the malignant B cells as revealed by analysis of cellular ATP levels and mitochondrial membrane integrity as well as caspase activation, respectively. As BAFF also plays a prominent role in autoimmune diseases, the BAFF-neutralizing antibody belimumab was developed and approved for treatment of systemic lupus erythematosus (SLE). When we employed belimumab in the context of CLL treatment with idelalisib, ibrutinib and venetoclax, BAFF neutralization was found to significantly increase the sensitivity of the leukemic cells to all three small molecule inhibitors. Notably, BAFF neutralization proved to be beneficial independently of clinical stage according to Binet and Rai or IgVH mutational status. Our results identify drug repurposing of belimumab for neutralization of BAFF to complement small molecule inhibitor treatment as a promising therapeutic approach in CLL that is presently undergoing clinical evaluation.

## 1. Introduction

Chronic lymphocytic leukemia (CLL) is the most common form of adult leukemia in Western countries [1]. In the last decades, patients suffering from CLL were treated with conventional chemotherapy in the vast majority of cases. After the introduction of the monoclonal CD20 antibody rituximab, which largely improved overall survival when added to chemotherapy, chemo-immunotherapy became the standard of care [2,3]. The increasing understanding of disease pathophysiology in CLL recently enabled the development of small molecule inhibitors specifically targeting molecular structures of central importance for prolonged survival of B cells. Three such compounds, the B cell receptor inhibitors ibrutinib and idelalisib as well as the Bcl-2 antagonist venetoclax have meanwhile been approved for CLL treatment and all profoundly improved long term survival of patients [4,5,6]. Nonetheless, many patients do not achieve complete remission upon treatment with these small molecule inhibitors, even in combination with CD20 antibodies, and disease progression was observed in 7% of idelalisib/rituximab-treated patients after six months, in 10% of ibrutinib/rituximab-treated patients after three years, and in 15% of venetoclax/ rituximab-treated patients after two years [5,7,8]. Disease progression after initial response is mainly caused by occurrence of resistance over the course of therapy [9,10,11,12]. 

A variety of different cytokines, chemokines and other factors are meanwhile recognized to protect CLL cells from spontaneous and drug-induced apoptosis. Available data have identified the tumor necrosis factor (TNF) family member B-cell activating factor (BAFF) to play a major role for survival of both healthy and malignant B cells [13,14,15,16]. BAFF binds to three different receptors: the ‘BAFF receptor’ (BAFFR), ‘transmembrane activator and cyclophilin ligand interactor’ (TACI), and ‘B-cell maturation antigen’ (BCMA). Pro-survival signals mediated by BAFF in B cells are mainly transduced via BAFFR, resulting in downstream activation of the NF-κB signaling pathway [16,17,18]. Several preclinical studies have meanwhile demonstrated that BAFF mediates pro-survival effects also in the context of B cell malignancies under therapy with currently approved treatment regimens [19,20,21,22,23,24]. 

From a clinical perspective, BAFF is mainly known for its role in autoimmune diseases: elevated BAFF serum levels have been detected in patients suffering from rheumatoid arthritis and SLE representing clinical pathologies that at least in part are caused by aberrant B cell activation [25,26]. In line, high serum levels of BAFF proved to be unfavorable prognostic factors due to their correlation with disease activity [27,28]. Based on its involvement in disease pathophysiology, BAFF neutralization using a monoclonal antibody termed belimumab has been established and was approved for the treatment of patients with SLE. We reported recently that BAFF protects primary CLL cells from rituximab-induced natural killer (NK) cell killing, and sensitivity of CLL cells to NK cell cytotoxicity could be restored by belimumab [24]. Here we demonstrate that BAFF impairs the therapeutic efficacy of the small molecule inhibitors idelalisib, ibrutinib and venetoclax and provide evidence that belimumab can resensitize CLL cells to treatment. The results of our study further serve as rationale for a multicentric phase II clinical study in which we will evaluate BAFF neutralization in combination with small molecule inhibitor application for treatment of CLL.

## 2. Results

### 2.1. BAFF Dose-Dependently Protects Primary CLL Cells from Treatment-Induced Cell Death

**Figure 1:**
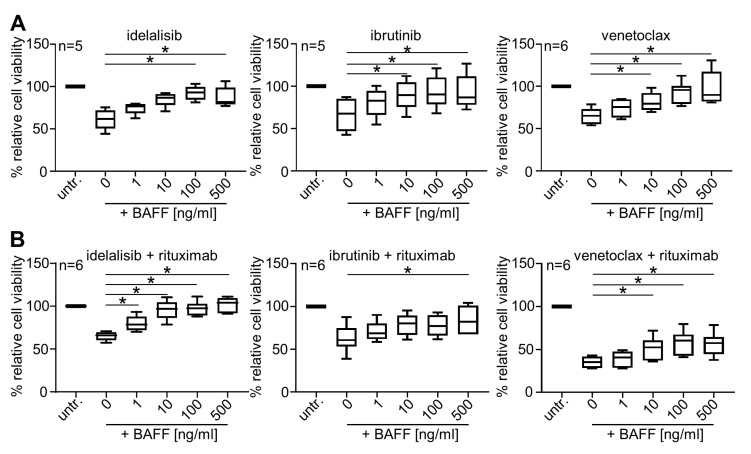

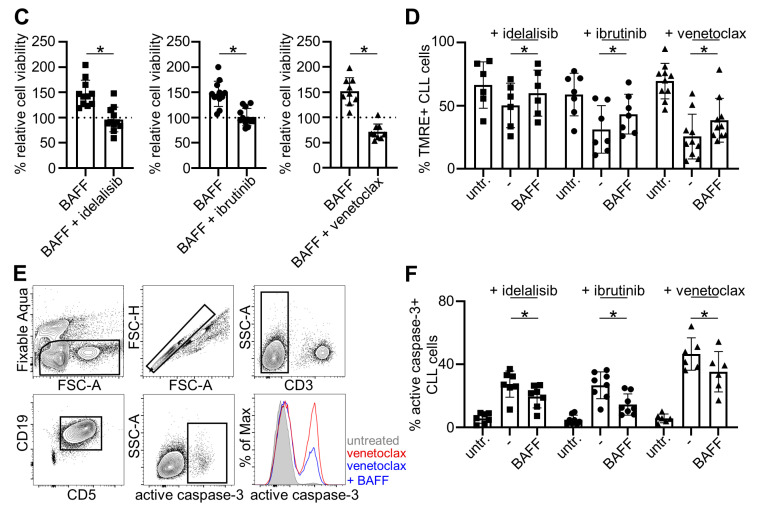
Protection of CLL cells from small molecule inhibitor-induced cell death by BAFF. (**A**,**B**) Peripheral blood mononuclear cells (PBMC) of CLL patients (*n* = as indicated) were exposed to idelalisib (500 nM), ibrutinib (500 nM) or venetoclax (2 nM) in combination with increasing concentrations of recombinant human BAFF (rhBAFF) in the absence (**A**) or presence (**B**) of rituximab (10 µg/mL). Cell viability was determined by measurement of ATP levels using the CTG assay. Results were normalized to the respective untreated samples, and relative viability was calculated. Boxes indicate the median and interquartile range, and whiskers represent data up to 1.5 × the interquartile range. RM one-way ANOVA with Tukey’s multiple comparisons tests were used for statistical analysis. (**C**) PBMC of CLL patients were exposed to rhBAFF in the presence or absence of idelalisib (*n* = 11), ibrutinib (*n* = 12) or venetoclax (*n* = 9) and then analyzed using CTG assays. Results were normalized to respective untreated samples without BAFF exposure (dotted line), and relative viability was calculated. For idelalisib and ibrutinib, a two-tailed *t*-test and for venetoclax a Wilcoxon matched-pairs signed rank test was used. (**D**) Mitochondrial membrane potential of primary CLL cells in the presence or absence of rhBAFF (500 ng/mL) was determined by staining with TMRE (Sigma, St. Louis, MO, USA) in 7-AAD-CD3- CD5+CD19+ cells using flow cytometry. Combined results of the percentages of TMRE+ primary CLL cells treated with idelalisib (10 µM, *n* = 7), ibrutinib (10 µM, *n* = 7) or venetoclax (5 nM, *n* = 10) are shown. For idelalisib and ibrutinib, a two-tailed *t*-test and for venetoclax a Wilcoxon matched-pairs signed rank test was used. (**E**,**F**) Active caspase-3 in Fixable Aqua-CD3-CD5+CD19+ cells in the presence or absence of rhBAFF (500 ng/mL) was determined intracellularly using flow cytometry. In (**E**), an example for the gating strategy is shown; (**F**) depicts combined results of the percentages of active caspase-3+ primary CLL cells treated with idelalisib (50 µM, *n* = 7), ibrutinib (10 µM, *n* = 8) or venetoclax (7.25 nM, *n* = 6). A two-tailed *t*-test was used for statistical analysis. Statistically significant differences (*p* < 0.05) are indicated by *.

In a first step, we determined suitable concentrations of the small molecule inhibitors idelalisib, ibrutinib and venetoclax for our in vitro studies based on plasma peak levels achieved in leukemia patients [29,30,31]. Then we incubated primary CLL cells with the selected concentration of idelalisib, ibrutinib or venetoclax in combination with increasing amounts of BAFF. Analyses of ATP levels using CellTiterGlo^®^ (CTG; Promega, Madison, WI, USA) assays revealed that BAFF dose-dependently protected CLL cells from drug-induced cell death (Figure 1A and data not shown). Notably, protective effects were already observed at concentrations as low as 1 ng/mL with more pronounced effects observed at higher levels. Of note, results of reports on BAFF levels (patho-)physiologically occurring in CLL differ substantially ranging from a median of 0.08 ng/mL [32] to 920 ng/mL [14]. Therefore and to allow for optimal elucidation of the effects of the small molecule inhibitors, 500 ng/mL BAFF was chosen as concentration for subsequent experiments.

Since CD20 antibodies are frequently included in current treatment regimens employing the small molecule inhibitors, we excluded that rituximab did influence the effect of BAFF on treatment susceptibility of the CLL cells (Figure 1B). Based on our results, we thus omitted addition of CD20 antibodies in subsequent experiments.

Notably, BAFF increased relative CLL cell viability in vitro also in the absence of small molecule inhibitors, which is in accordance with findings reported by Novak et al. [33] (Figure 1C). It remains unclear so far whether this finding is due to prevention of cell death caused by the in vitro culture conditions or caused by promotion of CLL cell survival as suggested by findings of Enzler et al. [34] obtained in animal models. 

Next we analyzed the influence of BAFF on treatment-induced apoptosis of the CLL cells in greater detail. To this end, we employed analysis of the mitochondrial membrane potential as detected by TMRE using flow cytometry. With all three small molecule inhibitors, we observed an induction of apoptosis as revealed by a decrease of the cell population with intact mitochondrial membrane potential, even though the extent varied between idelalisib, ibrutinib and venetoclax (Figure 1D). With all three compounds, presence of BAFF prevented the treatment-induced loss of mitochondrial membrane potential (Figure 1D).

To confirm these findings by analysis of a more downstream apoptotic event, we next analyzed how BAFF affected treatment-induced activation of caspase-3, a central executioner caspase which is basically responsible for cell degradation in the process of programmed cell death. To this end, primary CLL cells were exposed to idelalisib, ibrutinib or venetoclax in the presence or absence of BAFF followed by flow cytometry. The three small molecule inhibitors were found to induce caspase-3 activation to different extents, and in line with our previous results, BAFF reduced the processing of procaspase-3 into its active form in all cases (Figure 1E,F).

### 2.2. BAFF Mediates Anti-Apoptotic Effects in CLL Cells Irrespective of Clinical Stage or IgVH Mutational Status

Next, we studied whether the susceptibility to treatment with idelalisib, ibrutinib or venetoclax, or the protective effect of BAFF varied amongst patients depending on clinical features or molecular characteristics as reflected by the Binet as well as the Rai staging system and the IgVH mutational status, respectively [35,36,37]. Accordingly, the extent of cell death induced by the small molecule inhibitors and the protective effect of BAFF was determined using respective patient samples. 

**Figure 2:**
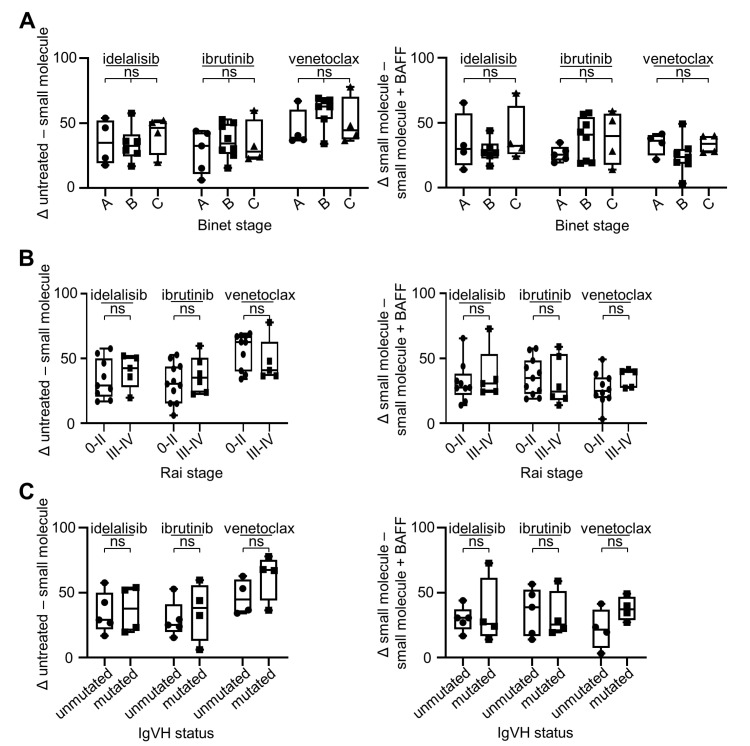
Clinical Binet stage, Rai stage and IgVH mutational status do not impact response to small molecule inhibitor treatment or survival-promoting effects of BAFF. PBMC from different CLL patients were exposed to idelalisib (500 nM), ibrutinib (500 nM) or venetoclax (2 nM) in the presence or absence of rhBAFF (500 ng/mL) and cell viability was determined by CTG assays. Results were normalized to the respective untreated samples and relative viability was calculated. The difference between the untreated sample and the small molecule inhibitor-treated sample (left side) and the difference between the respective small molecule inhibitor-treated sample alone and in combination with rhBAFF (right side) were analyzed. Results are depicted according to the clinical Binet stage (**A**), Rai stage (**B**) and IgVH mutational status (**C**). For statistical analyses, ordinary one-way ANOVA with Tukey’s multiple comparisons tests were performed in (**A**, left) for idelalisib (A vs. B *p* = 0.9766, A vs. C *p* = 0.8850, B vs. C *p* = 0.7557) and ibrutinib (A vs. B *p* = 0.7739, A vs. C *p* = 0.5810, B vs. C *p* = 0.9138), and a Kruskal-Wallis test with Dunn’s multiple comparisons test for venetoclax (A vs. B *p* = 0.8564, A vs. C *p* = 0.9279, B vs. C *p* > 0.9999) (idelalisib: Binet A *n* = 4, Binet B *n* = 5, Binet C *n* = 5; ibrutinib: Binet A *n* = 5, Binet B *n* = 7, Binet C *n* = 5; venetoclax: Binet A *n* = 4, Binet B *n* = 6, Binet C *n* = 5). In (**A**, right), a Kruskal-Wallis with Dunn’s multiple comparisons test was used for idelalisib (A vs. B *p* > 0.9999, A vs. C *p* > 0.9999, B vs. C *p* > 0.9999), and ordinary one-way ANOVA with Tukey’s multiple comparisons test for ibrutinib (A vs. B *p* = 0.4560, A vs. C *p* = 0.3997, B vs. C *p* = 0.9753) and venetoclax (A vs. B *p* = 0.5154, A vs. C *p* = 0.9090, B vs. C *p* = 0.7447). In (**B**, left), unpaired two-tailed *t*-tests were used for idelalisib (0-II vs. III-IV *p* = 0.4797) and ibrutinib (0-II vs. III-IV *p* = 0.4288), and for venetoclax a two-tailed Mann-Whitney test (0-II vs. III-IV *p* = 0.4396) (idelalisib: Rai 0-II *n* = 9, Rai III-IV *n* = 5; ibrutinib Rai 0-II *n* = 11, Rai III-IV *n* = 6; venetoclax Rai 0-II *n* = 10, Rai III-IV *n* = 5). In (**B**, right), a two-tailed Mann-Whitney test was used for idelalisib (0-II vs. III-IV *p* = 0.7972) and venetoclax (0-II vs. III-IV *p* = 0.0992), and an unpaired two-tailed *t*-test was performed for ibrutinib (0-II vs. III-IV *p* = 0.6485). In (**C**), unpaired two-tailed *t*-tests were used for idelalisib (left: unmutated vs. mutated *p* = 0.8180, right: unmutated vs. mutated *p* = 0.7128), ibrutinib (left: unmutated vs. mutated *p* = 0.6248, right: unmutated vs. mutated *p* = 0.8103) and venetoclax (left: unmutated vs. mutated *p* = 0.2135, right: unmutated vs. mutated *p* = 0.1358) (idelalisib: IgVH^unmut^ *n* = 5, IgVH^mut^ *n* = 4; ibrutinib: IgVH^unmut^ *n* = 5, IgVH^mut^ *n* = 4; venetoclax: IgVH^unmut^ *n* = 4, IgVH^mut^ *n* = 4). No statistically significant differences were observed, as indicated by “not significant (ns)”.

No relevant differences were observed when we employed the two clinical staging systems, neither with regard to the therapeutic effect nor the protective potential mediated by BAFF (Figure 2A,B). Likewise, no influence of the IgVH mutational status was observed (Figure 2C), which is consistent with previous reports from Herman et al. regarding the therapeutic effect of idelalisib and ibrutinib [38,39]. Overall this indicates that BAFF confers resistance to small molecule inhibitor treatment over the entire spectrum of disease.

### 2.3. Belimumab Abrogates Survival-Promoting Effects of BAFF

**Figure 3:**
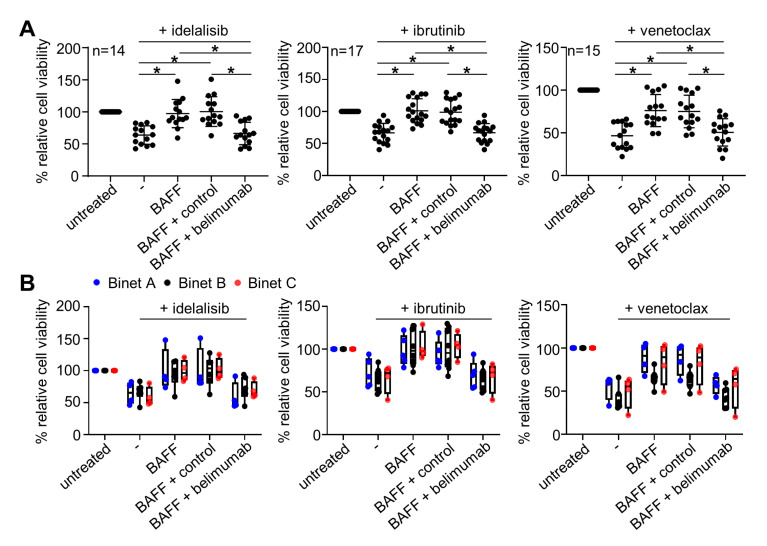

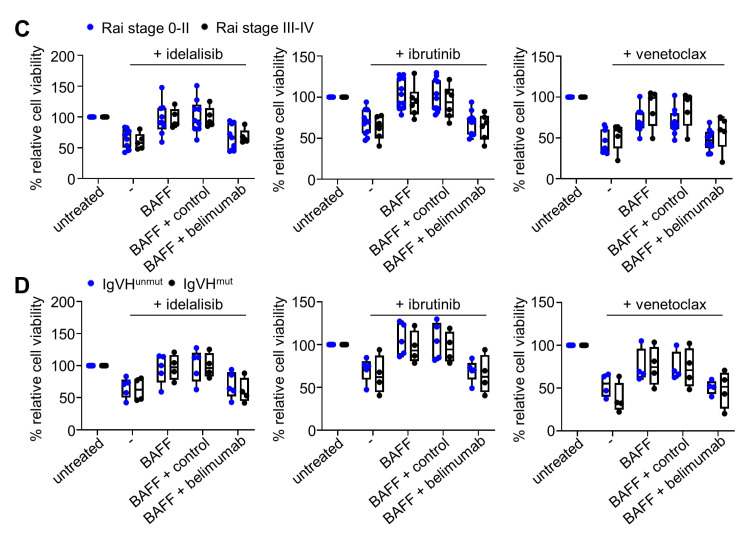
Neutralization of BAFF by belimumab sensitizes CLL cells to small molecule inhibitor treatment. (**A**) Cell viability of PBMC from CLL patients (n = as indicated) was determined using CTG assays after exposure to idelalisib (500 nM), ibrutinib (500 nM) or venetoclax (2 nM) in the presence or absence of rhBAFF (500 ng/mL), belimumab or control antibody (10 µg/mL each). Results were normalized to the respective untreated samples and relative viability was calculated. (**B**–**D**) Data sets depict effects according to clinical Binet stage (**B**), Rai stage (**C**) and IgVH mutational status (**D**). For statistical analyses, a RM one-way ANOVA with Tukey’s multiple comparisons test was performed in (**A**) for data sets of idelalisib, ibrutinib and venetoclax. In (**B**), for idelalisib, ibrutinib and venetoclax Kruskal-Wallis tests with Dunn’s multiple comparisons tests were used, and for venetoclax in addition ordinary one-way ANOVA with Tukey’s multiple comparisons tests (idelalisib: Binet A *n* = 4, Binet B *n* = 5, Binet C *n* = 5; ibrutinib: Binet A *n* = 5, Binet B *n* = 7, Binet C *n* = 5; venetoclax: Binet A *n* = 4, Binet B *n* = 6, Binet C *n* = 5). In (**C**), unpaired two-tailed *t*-tests were performed for idelalisib (Rai 0-II *n* = 9, Rai III-IV *n* = 5) and ibrutinib (Rai 0-II *n* = 11, Rai III-IV *n* = 6), and two-tailed Mann-Whitney tests for venetoclax (Rai 0-II *n* = 10, Rai III-IV *n* = 5). In (**D**), unpaired two-tailed *t*-tests were performed for idelalisib (IgVH^unmut^ *n* = 5, IgVH^mut^ *n* = 4), ibrutinib (IgVH^unmut^ *n* = 5, IgVH^mut^ *n* = 4), and venetoclax (IgVH^unmut^ *n* = 4, IgVH^mut^ *n* = 4). In (**A**), statistically significant differences (*p* < 0.05) are indicated by *. No statistically significant differences were observed in (**B**–**D**).

Next we exposed primary CLL cells to idelalisib, ibrutinib or venetoclax in the presence or absence of BAFF with or without belimumab or control antibody and determined CLL cell viability by CTG assays. In line with the results described above, treatment of primary CLL cells with the three small molecule inhibitors caused a relevant decrease of cell viability, and the CLL cells were rescued by BAFF. When these analyses were conducted in the presence of belimumab, we found that BAFF neutralization re-sensitized CLL cells to treatment with all small molecule inhibitors (Figure 3A). The effect of belimumab again held true over the entire spectrum of disease, as correlation of our in vitro results with clinical stage (Figure 3B,C) or IgVH mutational status (Figure 3D) did not reveal any statistically significant differences.

### 2.4. Belimumab Neutralizes Anti-Apoptotic Properties of BAFF

**Figure 4:**
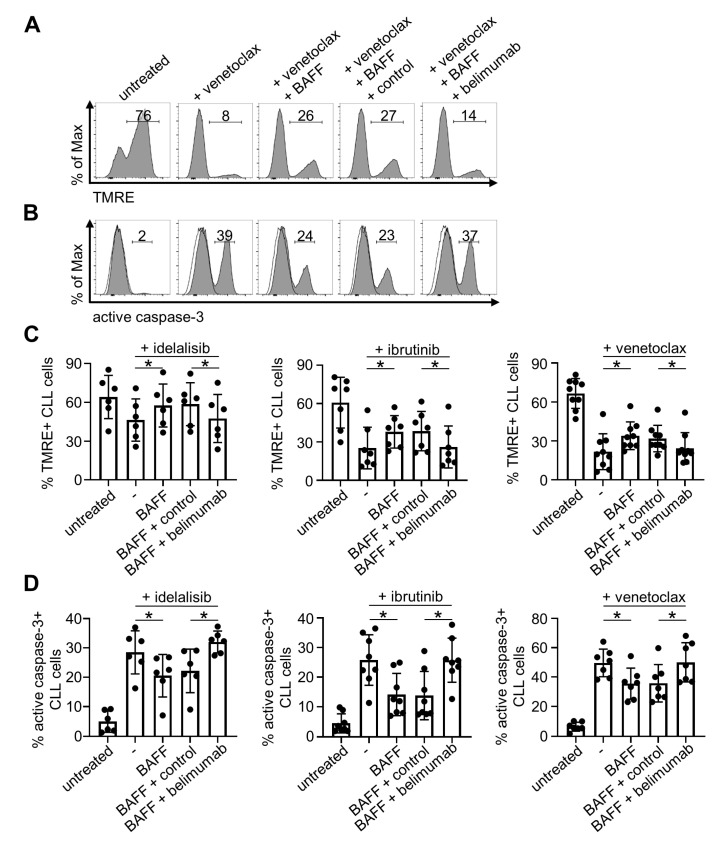
Neutralization of BAFF by belimumab re-sensitizes primary CLL cells to apoptosis induction by small molecule inhibitor treatment. Apoptosis in CLL cells was determined either by measurements of mitochondrial membrane potential with TMRE (**A**,**C**) or staining of active caspase-3 (**B**,**D**) using flow cytometry. (**A**,**C**) PBMC of CLL patients were incubated with idelalisib (10 µM, *n* = 6), ibrutinib (10 µM, *n* = 7) or venetoclax (5 nM, *n* = 9) in the presence or absence of rhBAFF (500 ng/mL), belimumab or control antibody (10 µg/mL each) followed by staining with CD3, CD5, CD19, 7-AAD and TMRE. Representative results in (**A**) are depicted for venetoclax showing the percentage of TMRE+ (= non-apoptotic) CLL cells, and combined results are shown in (**C**) for each small molecule inhibitor. For statistical analyses, a RM one-way ANOVA with Holm-Sidak’s multiple comparisons test was performed for idelalisib and ibrutinib, and Wilcoxon matched-pairs signed rank tests for venetoclax. (**B**,**D**) Activation of caspase-3 was analyzed in primary CLL cells exposed to idelalisib (50 µM, *n* = 6), ibrutinib (10 µM, *n* = 8) or venetoclax (7.25 nM, *n* = 7) in the presence or absence of rhBAFF (500 ng/mL), belimumab or control antibody (10 µg/mL each). Cells were stained with CD3, CD5, CD19, Fixable Aqua, an anti-active caspase-3 antibody and representative results are depicted in (**B**) as histograms, including the percentage of active caspase-3+ (= apoptotic) CLL cells and the isotype control signal (open histograms). Combined results are shown in (**D**). A paired *t*-test was performed for idelalisib, a Wilcoxon matched-pairs signed rank test for ibrutinib, and a RM one-way ANOVA with Tukey’s multiple comparisons tests for venetoclax. Statistically significant differences (*p* < 0.05) are indicated by *.

Finally, we aimed to determine whether and how BAFF neutralization by belimumab specifically affected apoptosis induction by the small molecule inhibitors. To this end, leukemic cells derived from different CLL patients were incubated with BAFF in the presence or absence of belimumab or control antibody and exposed to idelalisib, ibrutinib and venetoclax. Flow cytometric measurements revealed a statistically significant reduction of mitochondria membrane disruption in the presence of BAFF, and belimumab restored the susceptibility to mitochondria-dependent apoptosis with all compounds (Figure 4A,C). 

These results were confirmed by analyses of caspase-3 activation, which further revealed that belimumab prevents the anti-apoptotic effect of BAFF on CLL cells exposed to the small molecule inhibitors (Figure 4B,D). Together, these results demonstrate that the BAFF-neutralizing antibody belimumab might serve as an efficient tool for restoring susceptibility of CLL cells to all so far approved small molecule inhibitors.

## 3. Discussion

With exception of the few patients qualifying for allogeneic stem cell transplantation, CLL so far remains an incurable disease. This holds true despite the recent progress achieved by introduction of targeted treatment with the meanwhile approved small molecule inhibitors idelalisib, ibrutinib and venetoclax. All three compounds achieve superior outcomes with regard to overall and progression-free survival when compared to chemo-immunotherapy, the former standard of care in CLL [7,8,40]. However, alike observed for many other anti-cancer drugs, the potency of these small molecule inhibitors frequently declines in the course of treatment due to emerging resistance of the malignant cells. Beyond mutations that alter the structure of the target proteins and result in a lowered affinity to the inhibitors, a plethora of cell-mediated and soluble anti-apoptotic and pro-survival factors directly and indirectly contribute to treatment resistance [9,10,11,41,42,43,44].

Several studies have meanwhile provided evidence for the critical role of the TNF family member BAFF in CLL pathophysiology and therapy resistance [19,22,23,24,33]. BAFF mediates its effects via a variety of mechanisms, among which the NF-κB pathway represents the main route for pro-survival signal transduction resulting, among others, in upregulation of anti-apoptotic proteins like Bcl-2 and Mcl-1 [16,18,45]. The resulting imbalance between pro- and anti-apoptotic factors contributes to a longer lifetime of the malignant B cells and reportedly impairs susceptibility to chemotherapeutics [16,18,19,33,45,46,47]. 

We report here that BAFF impairs the efficacy of idelalisib, ibrutinib and venetoclax in CLL cells as revealed by analysis of treatment-induced cell death including mitochondria- and caspase-dependent apoptosis. Our findings are in line with findings of McWilliams et al. [22] and Sanchez-Lopez et al. [23], who reported similar effects in analyses involving ibrutinib and venetoclax, respectively. In contrast, Herman and coworkers [38,39] did not observe an effect of BAFF on the efficacy of idelalisib and ibrutinib. The reasons for the discrepancy of the findings of Herman et al. compared to our results and those of the other investigators remain so far unclear; certainly, differing experimental conditions, e.g., the employed BAFF concentrations, have contributed to the same. Notably, as already stated above conflicting findings have been reported with regard to the prevalence of BAFF in human serum. Menteşe et al. [32] described median soluble BAFF levels of 0.08 ng/mL in CLL patients, whereas median concentrations of 376 ng/mL (ranging from 93 to 8914 ng/mL) and 920 ng/mL (±54 ng/mL) were described by Molica et al. [48] and Haiat et al. [14], respectively. Of note, in all studies, levels in CLL patients were found to be lower than those of healthy controls [14,32,48]. This has been interpreted as evidence that BAFF may have been “depleted” from serum due to binding to its receptors expressed on CLL cells [49]. In addition, higher BAFF levels may prevail locally, e.g., in lymph nodes or proliferation centers. 

Further evidence for a protective effect of BAFF in CLL is provided by two studies in mice: Enzler et al. employed Eµ-TCL1xBAFF-Tg CLL mice presenting with high expression levels of BAFF and used BAFFR-Fc and BCMA-Fc fusion proteins to demonstrate that BAFF neutralization reduces leukemic burden in these animals [34,50]. In line, McWilliams et al. reported prolonged survival of SCID mice adoptively transferred with splenocytes from leukemic Eµ-TCL1 mice when an antibody that blocks murine BAFFR was applied to increase the therapeutic efficacy of ibrutinib [22]. 

As belimumab is not cross-reactive with mice [51], we employed primary human CLL cells ex vivo in our analyses which revealed that neutralization of human BAFF sensitizes patient CLL cells to treatment with idelalisib, ibrutinib and venetoclax. Of note, this beneficial effect was independent of the patients’ clinical stage according to Binet and Rai or IgVH mutational status. Although our in vitro experiments do not provide direct evidence to what extent endogenous BAFF provides survival signals to CLL cells, in particular when considering that, as discussed above, the extent of “bioactive” BAFF in CLL patients is yet unclear, these results demonstrate that BAFF confers resistance of CLL cells to all three small molecule inhibitors. Considering that B cells in humans reportedly require BAFF for survival it is reasonable to assume that blockade of BAFF in patients would sensitize CLL cells to treatment-induced cell death [52,53]. This in turn opens up a path for direct translation into clinical application: as belimumab is already approved for treatment of SLE, extensive data on the pharmacokinetics and safety profile in humans are readily available and allow for rapid repurposing of the antibody for CLL treatment [52,54]. This is even more since a phase I clinical study with CLL patients evaluating atacicept, which binds BAFF and its homologue APRIL has already provided evidence for the clinical safety of BAFF neutralization [55]. The finding that atacicept lacked therapeutic efficacy applied as monotherapy in this trial further supports our rationale to combine belimumab with application of small molecule inhibitors to increase the sensitivity of CLL cells to treatment. 

In a recent study we reported that BAFF also mediates resistance of CLL cells to treatment with rituximab and other CD20 antibodies [24]. These antibodies are integral components of CLL treatment that mediate their therapeutic efficacy in large part by inducing antibody-dependent cellular cytotoxicity of NK cells [56]. Our former study not only provided a functional explanation for the reportedly impaired efficacy of rituximab to induce NK cell reactivity in CLL, but also introduced BAFF neutralization as means to increase the efficacy of CD20 antibody treatment in this disease [24,57]. As combined application of idelalisib, ibrutinib or venetoclax with anti-CD20 antibodies has meanwhile been approved and rapidly changed treatment paradigms in CLL [58,59], we reason that belimumab may reinforce CLL treatment by a dual mechanism that comprises sensitization to both, (i) antibody-dependent cellular cytotoxicity induced by CD20 antibody treatment and (ii) induction of cell death by small molecule inhibitors. 

Among the three small molecule inhibitors, venetoclax appears particularly suitable for first clinical evaluation in a combinatorial approach with BAFF neutralization, because the combination of this Bcl-2 inhibitor and rituximab is the recommended second line treatment (category 1 according to NCCN Guidelines v.4.2020) employing targeted therapy together with CD20 antibody application. Addition of belimumab appears warranted as available data revealed a clonal evolution of CLL cells upon venetoclax treatment, despite its high efficacy [11,43,58,60]. This highlights the need for effective combinatorial approaches allowing for early elimination of minimal residual disease, prevention of development of secondary resistance and/or relapse and reduction of treatment time/toxicity. 

Based on the rationale outlined in this paper we have conceptualized a randomized clinical phase II study in which the safety and efficacy of combining subcutaneous application of belimumab with rituximab/venetoclax as second line treatment of relapsed/refractory CLL will be evaluated. The documents are presently submitted to the regulatory authorities, recruitment is expected to start within this year. This clinical trial will ultimately elucidate the physiological relevance of our results and whether BAFF neutralization can serve to further improve treatment options of patients.

## 4. Materials and Methods

### 4.1. Primary Material

Peripheral blood mononuclear cells (PBMC) were obtained from 36 CLL patients that had not undergone anti-cancer treatment for at least three months (mean CD5+/CD19+ fraction of 90.0% (range 81.1–96.0%), mean age 67.3 years (range 41–92), male (27), female (9); for additional characteristics see Table S1) at the University Hospital Tuebingen, Germany in the time from 2014–2019 after written informed consent and in accordance with the Helsinki protocol. The study was performed according to the guidelines of the local ethics committee (13/2007V). Mutational status of the variable region of the immunoglobulin heavy chain (IgVH) was assessed at time of initial diagnosis. Classification of the CLL patients according to the Binet or Rai staging system was based on clinical features and biomarkers at time of blood donation. For in vitro experiments, only PBMC samples with a CLL cell content of at least 80% were used.

### 4.2. Reagents

Recombinant human BAFF (rhBAFF) was from PeproTech (Rocky Hill, NJ, USA). Rituximab, trastuzumab and bevacizumab were from Roche (Basel, Switzerland), and belimumab was from GlaxoSmithKline (Brentford, UK). In neutralization experiments, binding of belimumab to rhBAFF was allowed for 45–60 min at room temperature prior to adding. Idelalisib and ibrutinib were from Selleckchem (Munich, Germany), and venetoclax was from Active Biochem LTD (Kowloon, Hong Kong). The antibodies CD5-APC, CD5-PE, CD19-FITC and anti-active caspase-3-AlexaFluor647 were purchased from BD Pharmingen (San Diego, CA, USA), CD3-eFluor450 was from eBioscience (San Diego, CA, USA). Rabbit isotype control-AlexaFluor647 was from Cell Signaling Technology (Danvers, MA, USA), LIVE/DEAD™ Fixable Aqua Dead Cell Stain Kit for 405 nm was from Invitrogen (Waltham, MA, USA), 7-aminoactinomycin D (7-AAD) and CD3-APCFire^TM^ 750 were from BioLegend (San Diego, CA, USA). Tetramethylrhodamine ethyl ester perchlorate (TMRE) was purchased from Sigma (St. Louis, MO, USA) and used at a concentration of 50 nM. Primary cells were cultivated in RPMI-1640 GlutaMAX^TM^ (Thermo Fisher Scientific, Waltham, MA, USA) supplemented with 10% fetal calf serum (Biochrom AG, Berlin, Germany) and 1% penicillin/streptomycin (Lonza, Basel, Switzerland).

### 4.3. Cell Viability Assay

PBMC from CLL patients were plated in a 96-well plate (Nunclon^TM^ Delta Surface, Thermo Fisher Scientific, Waltham, MA USA) at a density of 5 × 10^4^ per well and incubated with the indicated therapeutics in the presence or absence of rhBAFF, belimumab or control antibody (trastuzumab, bevacizumab). After 72 h, cell viability was measured using the CellTiterGlo^®^ Luminescent Cell Viability Assay (CTG) from Promega (Madison, WI, USA) according to the manufacturers’ instructions.

### 4.4. Analysis of the Mitochondrial Membrane Potential and Caspase-3 Activation

PBMC of different CLL patients were seeded at a density of 2 × 10^6^ per well in a 24-well plate (Corning Inc., Kennebunk, ME, USA) and pre-incubated with rhBAFF in the presence or absence of either control antibody or belimumab for 24 h. Then small molecule inhibitors were added in combination with rhBAFF, control antibody or belimumab as indicated before. 

Mitochondrial membrane potential was determined in CLL cells after 16 h (venetoclax), 48 h (ibrutinib) or 72 h (idelalisib) of incubation. After addition of CD3, CD5, CD19 antibodies and TMRE in combination with 7-AAD according to manufacturers’ instructions, patient samples with at least 30% TMRE+ cells were included in the analysis. 

For determination of caspase-3 activity in CLL cells after either 16 h (venetoclax) or 24 h of treatment with idelalisib or ibrutinib, PBMC were stained with CD3, CD5, CD19 and Fixable Aqua. Subsequent intracellular staining was performed using the Cytofix/Cytoperm buffer and Perm/wash solution (BD Biosciences, San Jose, CA, USA) with an anti-active caspase-3 or rabbit isotype control antibody according to manufacturers’ instructions. Patient samples with 10% or less basal caspase-3 activity as determined by analysis on either a FACSCanto II or a LSRFortessa cytometer (BD Pharmingen, San Diego, CA, USA) are shown.

### 4.5. Statistical Analysis

In general experiments were performed three times with various primary samples, and representative experiments as well as combined data are shown. For CTG assays, technical triplicates were performed enabling the calculation of average values and standard deviations. Statistical analyses were performed using GraphPad Prism 8 (GraphPad Software, San Diego, CA, USA). Normality distribution was tested using the Shapiro-Wilk normality test. For normally distributed data, *p*-values were calculated either by two-tailed *t*-tests, or repeated measures (RM) or ordinary one-way ANOVA and subsequent Tukey’s or Holm-Sidak’s multiple comparison tests. In case of non-normal distribution, *p*-values were calculated either by a Wilcoxon matched-pairs signed rank test, a two-tailed Mann-Whitney test or a Kruskal-Wallis test followed by Dunn’s correction for multiple comparisons. Where indicated, statistically significant (*p* < 0.05) differences between two groups are marked by “*”. Boxes depict the 25th to 75th percentiles, whiskers extend to the minimum and up to the maximum value, centre values represent mean, error bars represent standard deviation (s.d.) if not declared otherwise.

## 5. Conclusions

Here, we report that drug repurposing of the clinically available BAFF-neutralizing antibody belimumab, which is approved for treatment of SLE, re-sensitized primary CLL cells to the small molecule inhibitors idelalisib, ibrutinib and venetoclax. Based on our findings, we conclude that the combined application of belimumab with rituximab/venetoclax for treatment of relapsed/refractory CLL constitutes a promising therapeutic approach to elucidate whether neutralization of BAFF can serve to further improve treatment options of patients.

## Supplementary Materials

The following are available online at https://www.mdpi.com/2072-6694/12/10/2725/s1, Table S1: Patient characteristics.
