# Supplementary material for: Neutralization of B-Cell Activating Factor (BAFF) by Belimumab Reinforces Small Molecule Inhibitor Treatment in Chronic Lymphocytic Leukemia
Source: Cancers (Basel). 2020 Sep 23;12(10):2725. doi: 10.3390/cancers12102725 (PMC7598196; doi:10.3390/cancers12102725)
Supplement: Supplementary file 1 [file cancers-12-02725-s001.pdf]

cancers

MDPI

Supplementary Materials: Neutralization of B-cell activating factor (BAFF) by belimumab reinforces small molecule inhibitor treatment in chronic lymphocytic leukemia

Claudia Tandler, Moritz Schmidt, Jonas S. Heitmann, Julia Hierold, Jonas Schmidt, Pascal Schneider, Daniela Dörfel, Juliane Walz and Helmut R. Salih

Table 1. Patient characteristics.

Sex	Age	Rai stage	Binet stage	Hb	WBC	Plt	CD5+/CD19+	IgVH status	del 11q22.3	del 13q14	trisomy 12
f	59	III	C	9.5	347.0	158	94.3	unmutated	n.a.	n.a.	n.a.
f	52	I	B	12.2	90.9	110	90.9	n.a.	n.a.	n.a.	n.a.
f	81	III	C	9.1	156.4	129	96.0	unmutated	n.a.	n.a.	n.a.
f	79	I	B	11.4	38.8	145	90.3	n.a.	n.a.	n.a.	n.a.
f	53	III	B	10.6	220.4	222	81.1	mutated	+	n.a.	n.a.
f	82	I	A	11.9	130.8	166	92.6	n.a.	n.a.	n.a.	n.a.
f	50	II	B	11.1	58.6	138	95.4	mutated	n.a.	n.a.	n.a.
f	76	IV	C	9.5	64.0	93	90.8	n.a	n.a.	n.a.	n.a.
f	51	III	A	10.0	48.3	155	90.2	unmutated	n.a.	n.a.	n.a.
m	54	II	A	14.4	86.9	186	85.5	mutated	n.a.	n.a.	n.a.
m	53	IV	C	14.1	59.0	96	94.1	mutated	n.a.	+	n.a.
m	76	IV	C	13.5	55.0	62	93.5	n.a.	n.a.	+	n.a.
m	71	II	B	11.2	264.5	103	90.3	unmutated	n.a.	+	n.a.
m	75	III	B	10.0	238.6	246	88.8	n.a.	n.a.	+	+
m	81	III	C	9.4	86.1	107	95.1	mutated	n.a.	n.a.	n.a.
m	61	I	B	13.9	73.9	173	82.5	n.a.	n.a.	n.a.	n.a.
m	92	IV	C	9.6	123.6	83	86.4	n.a.	n.a.	n.a.	n.a.
m	41	I	B	13.1	124.5	155	86.7	mutated	n.a.	+	n.a.
m	78	IV	C	12.1	181.1	92	81.5	n.a.	n.a.	n.a.	n.a.
m	72	IV	C	8.6	824.0	84	88.4	unmutated	n.a.	n.a.	n.a.
m	67	0	A	13.3	70.7	155	86.3	mutated	n.a.	n.a.	n.a.
m	66	I	A	13.4	155.6	127	95.6	unmutated	+	n.a.	n.a.
m	73	IV	C	11.5	299.6	91	94.4	unmutated	+	n.a.	n.a.
m	73	I	A	13.7	88.8	116	88.7	n.a.	n.a.	n.a.	n.a.
m	59	0	A	14.7	57.8	148	84.7	n.a.	n.a.	n.a.	n.a.
m	69	0	A	13.1	26.8	188	81.5	n.a.	n.a.	n.a.	n.a.
m	91	III	C	9.9	160.6	179	95.6	n.a.	n.a.	n.a.	n.a.
m	48	II	B	13.5	111.7	167	94.2	unmutated	+	+	n.a.
m	89	II	A	13.1	155.6	132	87.5	n.a.	n.a.	n.a.	n.a.
m	69	III	B	10.5	537.4	203	89.6	n.a.	+	n.a.	n.a.
m	67	IV	C	7.6	59.2	93	92.4	n.a.	n.a.	n.a.	n.a.
m	55	II	B	11.8	227.9	119	91.9	unmutated	n.a.	+	n.a.
m	57	I	B	14.7	96.7	181	93.8	unmutated	n.a.	+	n.a.
m	59	II	B	15.9	147.3	187	91.1	unmutated	+	n.a.	n.a.

m	68	III	C	9.4	223.9	227	89.9	mutated	n.a.	n.a.	+
m	76	II	A	13.8	75.0	206	87.8	mutated	n.a.	n.a.	n.a.

Abbreviations: del, deletion; Hb, hemoglobin [g/dl]; f, female; IgVH, immunoglobulin heavy chain variable region gene; n.a., not assessed; m, male; Plt, platelets. [x10^6/mL]; WBC, white blood count [x10^6/mL]. Reference values: Hb: female 12 - 16 g/dl, male 13 - 17 g/dl, Plt: 140 000 - 345 000/μL, WBC: 3 800 - 10 500/μL.

© 2020 by the authors. Licensee MDPI, Basel, Switzerland. This article is an open access article distributed under the terms and conditions of the Creative Commons Attribution (CC BY) license (http://creativecommons.org/licenses/by/4.0/).
